# Huntington's Disease as Neurodevelopmental Disorder: Altered Chromatin Regulation, Coding, and Non-Coding RNA Transcription

**DOI:** 10.3389/fnins.2015.00509

**Published:** 2016-01-13

**Authors:** Emanuela Kerschbamer, Marta Biagioli

**Affiliations:** NeuroEpigenetics Laboratory, Centre for Integrative Biology, University of TrentoTrento, Italy

**Keywords:** Huntington's Disease, neurodegenerative disorders, neurodevelopmental disorders, chromatin regulation, coding and non-coding RNA transcription

Huntington's disease (HD) is a monogenic autosomal dominant, fatal disorder due to CAG trinucleotide expansion in exon 1 of the HD gene (*HTT*) (The Huntington's Disease Collaborative Research Group, 1993; Figure 1A). Nowadays, there is no cure or effective treatment for the disease which presents with motor, cognitive and psychiatric dysfunction. Although typically conceived as a “neurodegenerative disease,” mainly affecting the GABAergic medium-sized spiny neurons (MSN) of the striatum and deep layers of the cortex (Rosas et al., 2003), an increasing body of evidence has surfaced indicating that abnormal neurodevelopment might also have a crucial role in HD (Mehler and Gokhan, 2000, 2001; Humbert, 2010). Neurodegenerative diseases have been classically defined as chronic and progressive disorders of the nervous system affecting neurologic and behavioral functions, which start with specific biochemical changes that ultimately lead to distinct histopathologic and clinical syndromes. On the contrary, neurodevelopmental disorders result from an anomaly of brain maturation, during fetal or early postnatal life, which is postulated to alter the structure and/or function of neuronal and synaptic populations (Harrison, 1995).

**Figure 1:**
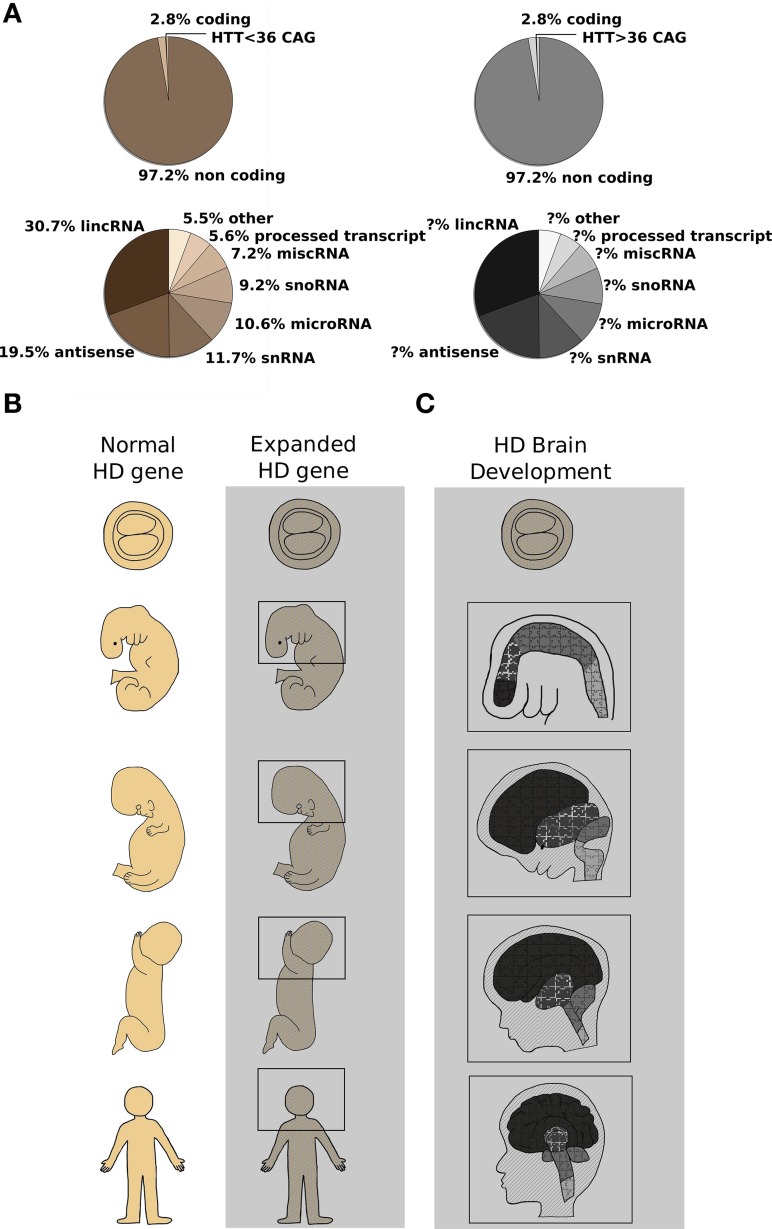
**CAG expansion in the human ***HTT*** gene leads to Huntington's Disease (HD): possible contribution of altered non-coding RNA transcription and aberrant developmental processes are described**. **(A)** Top pie charts report the proportion of coding and noncoding RNAs in the human genome (Alexander et al., 2010). Highlighted in the coding portion of the genome, the Huntington's Disease gene (*HTT*). Wild type or expanded CAG lengths are indicated by sepia and gray colors, respectively. Bottom pie charts summarize the relative contribution of single classes of non-coding RNAs (Baker, 2011) to normal (sepia) or diseased (gray) organism physiology. The contribution of non-coding RNA transcription to HD process is still not fully dissected. **(B)** Schematic representation of human development at different stages [blastocyst (5 days) and 25 days embryos, 100 days and 5 months fetuses and adult organism] is shown. Normal (sepia) and mutant huntingtin [expanded *HTT* gene] (grayscale) expression is found in the whole organism at any given developmental stage (MacDonald et al., 2003; Gusella and MacDonald, 2006). Molecular mechanisms (miRNAs, lncRNAs, alterative splicing, histone modifications and chromatin remodeling) acting since conception in the organism bearing the mutation are represented by the gray area. **(C)** A simplified representation of HD brain development is shown. Developmental stages as in **(B)**. Different color's shades denote specific brain regions such as telencephalon, diencephalon, midbrain, and hindbrain which develop differently to originate adult brain structures. Light gray lines across the head designate coronal sections unveiling inner brain organization, while puzzle pieces represent various cell types constituting each brain district. It is evident that the presence of mutant huntingtin (expanded *HTT* gene) places any cell of the organism—and of the brain in particular—in a “different biological state” with subtle cellular and tissue development alterations that culminate in the overt symptoms appearing in adult life. The changes induced by the expression of CAG-repeat *HTT* expansion compromise MSN-generating stem cells specification and prime MSN to adult neuronal degeneration (Molero et al., 2009; Humbert, 2010).

This opinion piece discusses the existing evidences from HD patients and HD model systems for a neurodevelopmental component to the neurodegenerative processes of Huntington's Disease pathophysiology.

The first and most striking observations come from multi-national neuropathological and neuroimaging studies of prodromal HD, reporting clear brain changes decades prior to onset of disease: specifically, smaller head circumference (Lee et al., 2012), caudate and putamen atrophy (Nopoulos et al., 2007; Paulsen et al., 2010), striatal and cortical white matter abnormalities including increased density of oligodendrocytes (Gómez-Tortosa et al., 2001) and cortical thinning (Nopoulos et al., 2007) were described. Moreover, subtle but significant defects in cognitive (Solomon et al., 2007), behavioral (Duff et al., 2007), and motor function (Hinton et al., 2007) have been identified in prodromal HD subjects long before a clinical diagnosis. While these observations might be linked to early neuronal degeneration, the other plausible interpretation is that they represent manifestations of subtle altered development that prime specific areas and neuronal subpopulations of the brain to later catastrophic consequences (Figure 1).

Further indications come from studies of mouse models with inactivation of the HD orthologue gene *Htt*. Huntingtin protein, in fact, is expressed in all cell types of the body, at all developmental stages and it has crucial roles during development and neurogenesis (Figure 1B). Parallel studies have shown that complete lack of huntingtin in mice results in embryonic lethality with developmental arrest just post gastrulation (Duyao et al., 1995; Zeitlin et al., 1995), while severe reduction of huntingtin levels in heterozygous, *Htt* hypomorphic mice or *Htt* conditional knock-out neuronal subpopulations manifests in abnormal brain development, cognitive and motor abnormalities (Nasir et al., 1995; White et al., 1997; Auerbach et al., 2001; Godin et al., 2010). Given this key role of wild-type huntingtin during development, it is not surprising that studies on genetic engineered mice expressing *Htt* CAG-expansion pathogenic mutations show subtle molecular and structural deficits that portend altered developmental processes and precede the occurrence of neurological symptoms and signs of cell death (Luthi-Carter et al., 2000; Wheeler et al., 2000; Fossale et al., 2011). Specifically, even in *Hdh* knock-in mice, representing a faithful genetic replica of the human HD mutation [expressing full-length mutant huntingtin at endogenous levels from the native promoter at the *Htt* locus], although usually presenting milder molecular and behavioral phenotypes compared to HD transgenic and fragment lines, one single copy of the mutant *Htt* allele is sufficient to cause nuclear accumulation of full-length mutant huntingtin in MSN as early as 3 weeks of age (Wheeler et al., 2000), molecular changes in pathways related to energy metabolism and cAMP levels, RNA metabolism (Gines et al., 2003) and measurable transcription/translation dysfunctions (Fossale et al., 2011) at 3 and 10 weeks of age, well before the onset of any overt pathological alteration. Strikingly, in the same *Hdh* Q111 knock-in HD mouse model, deregulation of the temporal and spatial profiles of striatal neurogenesis [delayed cell cycle exit and transient aberrant expression of pluripotency markers in MSN-generating neural stem cells] (Molero et al., 2009) may expose striatal precursors to inappropriate molecular cues driving them into aberrant programs of neuronal differentiation, thus reinforcing the notion that altered neurodevelopment might forecast later MSN susceptibility in HD (Figure 1C).

Additional consistent observations emerged from studies using mouse embryonic stem cells and neural committed progenitors bearing *Htt* CAG-expansion mutations (Jacobsen et al., 2011; Conforti et al., 2013; Biagioli et al., 2015) as well as patient-derived induced pluripotent stem cells (iPSCs) (The HD iPSC Consortium, 2012), showing measurable molecular differences and a series of expanded CAG-associated phenotypes that point toward a central role of wild-type and mutant huntingtin in signal transduction, axonal guidance, synaptic transmission and neurodevelopment. Specifically, unbiased “- omics” analyses, probing transcriptomic, chromatin modifications and DNA methylation status in these cells and their neuronal derivatives, support the hypothesis that wild-type and mutant huntingtin might affect key chromatin regulators such as DNA and histone methyl transferases, and demethylases [Polycomb Repressive Complex 2 (PRC2), Mixed Lineage Leukemia 1–4, JARID1, REST, HYPB-SETD2] (Lee et al., 2013; Ng et al., 2013; Biagioli et al., 2015). In fact, a growing body of evidence suggests that alterations of epigenetic modifications constitute a basic molecular mechanism caused by the HD mutation and responsible for early features of the pathological process. Strikingly, most of these epigenetic regulators, i.e., Polycomb Complexes, but also other histone post-transcriptional modifications enzymes, have important roles during transition from pluripotency to differentiation and neural development, in particular (Jepsen et al., 2007). While the exact mechanisms of this “huntingtin chromatin function” are not entirely known, some evidence has accumulated suggesting that wild-type and CAG-expanded huntingtin may interact, directly or indirectly, with epigenetic co-regulators and alter their activity (Seong et al., 2010). Interestingly, the DNA methylation pattern of several promoter regions of genes involved with pluripotency and neural differentiation (*Sox2, Pax6, Nes*) was found significantly reduced in presence of mutant huntingtin (Ng et al., 2013), while the histone methylation status of a class of developmentally-regulated bivalent promoters associated with “Regulation of Neurogenesis” and “Stress Signaling and Apoptosis” was altered following the expression of CAG-expanded *Htt* alleles (Biagioli et al., 2015). Thus, huntingtin protein might subtly but consistently alter different aspects of chromatin regulation and transcription during neural development and specification, explaining the pleiotropic, subtle yet deleterious, effects of mutant huntingtin observed throughout the life of an HD individual (Figures 1B,C).

Parallel to alterations in the coding portion of the genome, a growing number of studies is recently showing deregulation of different classes of non-coding RNAs (ncRNAs)—microRNAs (miRNAs), long non-coding RNAs (lncRNAs), piwi-interacting RNAs (piRNAs), circular RNAs (circRNAs)—suggesting that they may have a relevant impact on disease onset/progression. A large percentage of ncRNAs physically interact with various chromatin regulatory proteins, including PRC2, and other “readers/writers, and erasers” of chromatin modifications, thus activating or repressing gene expression via a chromatin recruitment mechanism. The first, intriguing example of such regulatory network is the mammalian X-chromosome inactivation, where the finely regulated expression of *Xist* lncRNA is able to recruit PRC2 to the specific genomic location of the silenced X chromosome to mediate transcriptional repression. Recently, systematic studies exploring ncRNA function have shown their involvement in many biological processes also related to embryonic development and neurogenesis (Qureshi and Mehler, 2012, Sauvageau et al., 2013). Particularly, several miRNAs, but also lncRNAs have been reported to be enriched in the brain and have key roles during transition from neural commitment to terminal differentiation (Makeyev et al., 2007; Sauvageau et al., 2013). Importantly, miRNAs might also target neuronal-specific transcriptional regulators (i.e., REST and co-REST) as well as brain-enriched alternative-splicing factors, thus affecting synaptic activity and neuronal function (Makeyev et al., 2007; Packer et al., 2008). Mutant huntingtin expression has been correlated with alterations in REST cellular localization and dysregulation of REST-regulated miRNAs (miR-9 and miR-124) levels (Packer et al., 2008). Specifically, miR-9 and miR-124 levels were shown to be reduced in the cortical areas of HD *post mortem* brains where genes related to neurogenesis were highly overrepresented (Hodges et al., 2006). Moreover, a recent genome-wide screen of miRNAs in *post mortem* brains highlighted miRNAs that were differentially expressed between normal and HD subjects (Hoss et al., 2014). The vast majority of these miRNAs are highly connected with HOX clusters, a well-studied family of transcription factors, involved in early brain development, contributing to anterior-posterior positional establishment. HOX genes and HOX-related miRNAs are tightly regulated by PRC2 proteins and are found to be altered following mutant huntingtin expression (Seong et al., 2010). Although the role of development-specific miRNAs and HOX genes in the adult HD brain is still unclear, one interpretation might suggest aberrant brain development following mutant huntingtin expression, while, a second plausible explanation might invoke a reactivation of developmental programs in the later phases of HD degenerative process where surviving and neurogenic hints try to counteract generalized neuronal loss.

MiRNAs, however, are not the only class of ncRNAs with developmental potential to be dysregulated in HD. Several additional studies, in fact, report altered expression levels of known lncRNAs *TUG1, NEAT1, MEG3,* and *DGCR5* in the brains of HD patients (Johnson, 2012). *MEG3*, in particular, is reported to be a REST target with a dynamic expression during development of the nervous system and associated with PRC2 chromatin regulator, thus supporting a role for chromatin regulation, non-coding transcription and neurodevelopment in HD pathogenesis.

Other classes of ncRNA (pi-RNAs and circRNAs), associated with chromatin remodeling factors and epigenetic modulators, have a clear role during neural development and differentiation, however their regulation upon expression of mutant huntingtin has not been analyzed yet.

The analysis of non-coding RNA transcription and its interplay with chromatin regulation in the context of human neurological disease presents several challenges especially related to the complexity of ncRNAs in the brain. In fact, the annotation, identification and functional characterization of ncRNAs continue to be puzzling because of the lack of a complete understanding of functional domains, their generally low expression levels, and the poor knowledge of their regulatory regions (Esteller, 2011). International initiatives such as the Encyclopedia of DNA Elements (ENCODE) or the generation of knock-out mice for specific ncRNAs (Sauvageau et al., 2013, Goff et al., 2015) represent the first effort to understand the functional relevance of ncRNAs in the organism (and brain) physiology and disease. Additional studies in this direction as well as new and powerful genomic and bioinformatic tools will be crucial to dissect the complexity of ncRNA brain transcriptome to understand how the interaction between epigenome and transcriptome will contribute to disease onset and progression and shed light on possible new paths to therapeutic intervention.

In conclusion, it's time to rethink the simple view of Huntington's Disease as a merely neurodegenerative disorder to include the idea that expression of the single copy of the CAG-expanded *HTT* allele (Figure 1A) is also sufficient to subtly alter development from conception. The “HD organism” is, at any developmental time point, different from the “not-HD counterpart” (Figures 1B,C). Like in a puzzle where the *HTT*-CAG mutation forces pieces to fit where they do not belong, similarly subtle developmental differences may give rise to a different panoply of adequately functioning units which may last for a long time but ultimately not allow the same flexibility with aging, thus aberrant neurodevelopment might lead to disorganization and dysconnectivity of neurons and subsequent susceptibility to neurodegenerative processes (Figures 1B,C). Essential corollaries of this model are not only new approaches to the understanding of the altered pathologic process, but also new strategies of therapeutic intervention/prevention. In fact, the recent identification of genetic factors that are able to modify the age of onset of HD patients, indicate that the HD pathologic process is modifiable prior to clinical diagnosis (Genetic Modifiers of Huntington's Disease (GeM-HD) Consortium, 2015), thus representing a proof-of-principle of a strategy that is predicated upon the idea that intervening before onset of clinical signs is likely to be most effective to finally ameliorate patients quality of life and/or life expectancy.

## Author contributions

EK made substantial contributions to conception and design of the work and participated in drafting the manuscript; MB conceived and supervised the project and wrote the paper. Authors read and gave final approval of the manuscript version version to be submitted.

### Conflict of interest statement

The authors declare that the research was conducted in the absence of any commercial or financial relationships that could be construed as a potential conflict of interest.
